# Interprofessional team member’s satisfaction: a mixed methods study of a Chilean hospital

**DOI:** 10.1186/s12960-018-0290-z

**Published:** 2018-07-11

**Authors:** Pilar Espinoza, Marina Peduzzi, Heloise F. Agreli, Melissa A. Sutherland

**Affiliations:** 1 School of Nursing, Andes University of Chile, Moseñor Alvaro de Portillo 12455, Las Condes, Santiago Chile; 2 School of Nursing, Department of Professional Guidance, University of São Paulo, Av. Dr. Enéas de Carvalho Aguiar 419, Cerqueira Cesar, São Paulo, SP 05403-000 Brazil; 3 Catherine McAuley School of Nursing & Midwifery, University College Cork. Brookfield, College Rd,University College, Cork, T12 K8A Ireland; 4 William F. Connell School of Nursing, Boston College, 140 Commonwealth Ave, Chestnut Hill, MA 02467 United States of America

**Keywords:** Interdisciplinary teams, Healthcare teams, Team work, Satisfaction with the team, Transformational leadership, Team climate, Mixed methods, Equipos interdisciplinarios, equipos de salud, trabajo en equipo, satisfacción con el equipo, liderazgo transformacional, clima de equipo, métodos mixtos

## Abstract

**Introduction:**

The health organizations of today are highly complex and specialized. Given this scenario, there is a need for health professionals to work collaboratively within interprofessional work teams to ensure quality and safe care. To strengthen interprofessional teamwork, it is imperative that health organizations enhance strategic human resources management by promoting team member satisfaction.

**Objective:**

To analyze the satisfaction of members in interprofessional teams and to explore interpersonal relationships, leadership, and team climate in a hospital context.

**Methodology:**

This study is an explanatory sequential mixed methods (quantitative/qualitative) study of 53 teams (409 professionals) at a university hospital in Santiago, Chile. The first phase involved quantitative surveys with team members examining team satisfaction, transformational leadership, and team climate. Social network analysis was used to identify interactions among team members (cohesion and centrality). The second phase involved interviews with 15 professionals belonging to teams with the highest and lowest team satisfaction scores. Findings of both phases were integrated.

**Results:**

Significant associations were found among variables, and the linear regression model showed that team climate (*β* = 0.26) was a better predictor of team satisfaction than team leadership (*β* = 0.17). Registered nurse was perceived as the profession with the highest score on the transformational leadership measure (mean = 64), followed by the physician (mean = 33). Team networks with the highest and lowest score of team satisfaction showed differences in cohesion and centrality measures. Analysis of interviews identified five themes: attributes of interprofessional work; collaboration, communication, and social interaction; interprofessional team innovation; shared leadership; and interpersonal relationship interface work/social. Integration of findings revealed that team member satisfaction requires participation and communication, common goals and commitment for patient-centered care, clear roles and objectives to support collaborative work, and the presence of a transformational leader to strengthen well-being, dialog, and innovation.

**Conclusions:**

Results have the potential to contribute to the planning and decision-making in the field of human resources, providing elements to promote the management of health teams and support team member satisfaction. In turn, this could lead to job permanence especially where the local health needs are more urgent.

## Resumen

**Introducción:**

Las organizaciones de salud son altamente complejas y especializadas. En este escenario es necesario que los profesionales de salud trabajen colaborativamente en equipos de trabajo interprofesionales, asegurando un cuidado de calidad y seguro. Para fortalecer el trabajo en equipo interprofesional, es imperativo mejorar la gestión estratégica de recursos humanos en organizaciones de salud, promoviendo la satisfacción de sus miembros.

**Objetivo:**

Analizar la satisfacción de los miembros de equipos interprofesionales, explorando sus relaciones interpersonales, liderazgo y clima de equipo en un contexto hospitalario.

**Metodología:**

Estudio de métodos mixtos secuencial explicatorio (cuantitativo-cualitativo) en 53 equipos (409 profesionales) de un hospital universitario en Santiago, Chile. Primera fase incluyó encuestas a los miembros de equipos para examinar satisfacción con el equipo, liderazgo transformacional y clima. Utilizando análisis de redes sociales para identificar interacciones entre sus miembros (densidad y cohesión). Segunda fase incorpora 15 entrevistas a profesionales de equipos con los puntaje más altos y bajos de satisfacción. Integración de los resultados de ambas fases.

**Resultados:**

Asociaciones significativas entre las variables y un modelo de regresión lineal que reveló que el clima de equipo (*β* = 0.26) es un mejor predictor de la satisfacción con el equipo, que el liderazgo transformacional (*β* = 0.17). Enfermería fue el profesional con el más alto puntaje en liderazgo transformacional (media = 64), seguido por el medico (media = 33). Las redes sociales de los equipos con puntajes extremos de satisfacción mostraron diferencias en cohesión y centralidad. El análisis de las entrevistas entrego cinco temas: *atributos del trabajo interprofesional; colaboración, comunicación e interacción social; innovación en equipo interprofesional; liderazgo compartido; interface entre relaciones interpersonales de trabajo y social.* Integración de los resultados reveló que la satisfacción requiere participación y comunicación, metas comunes y compromiso con un cuidado centrado en el paciente, claridad de roles y responsabilidades para trabajar colaborativamente y la presencia de liderazgo transformacional para fortalecer el bienestar, diálogo e innovación.

**Conclusión:**

Los resultados tienen el potencial de contribuir a la planificación y toma de decisiones en el área de recursos humanos en salud con elementos que promuevan la administración de equipos de salud apoyando la satisfacción de sus miembros. Pudiendo llevar a permanencia laboral especialmente donde las necesidades locales de salud son más urgentes.

## Background

Today’s health organizations are highly complex and specialized. The knowledge and skills necessary to effectively and efficiently meet the goals of the health organization are changing constantly, and are also associated with greater expectations and demands from patients. Given this situation, it is essential that health professionals collaborate within interprofessional work teams, to improve performance and enhance the quality and safety of care [1, 2].

The World Health Organization (WHO) through the Global Strategy for Human Resources for Health (HRH) 2030 calls on countries to “adopt a different paradigm in the management of health personnel and assume obligations for the optimization of their performance, through evidence-based policies and practices that promote collaborative interprofessional teamwork, job opportunities and ongoing training, innovation and the use of scientific evidence” [3].

In health care settings, work is characterized by its variability and complexity. Within this context, there is a need for interprofessional teams with the skills and knowledge necessary to respond to the changing environment and complex needs of patients [4]. Effective teamwork can optimize patient care and promote the job satisfaction and retention of its members [5]. Successful clinical outcomes have been associated with team members’ interpersonal relationships, communication, and cooperation. This is turn, can result in the creation of a stimulating work environment [6, 7].

Job satisfaction is relevant not only in terms of the well-being of people, but in relation to work productivity and quality [8]. Job satisfaction has also been associated with interprofessional collaboration, communication, and professional commitment [4, 5, 9]. Specifically, team satisfaction is the members’ attitudes towards the team. It is the extent to which team members have a positive and pleasant feeling that encourages them to work in the same team again [10, 11]. Team satisfaction is reflected in shared decision-making, [11] effective team functioning, [12, 13], and team stability [14]. On the other hand, dissatisfaction is a predictor of absenteeism, job change, and abandonment [15] and may result in poor work processes, inconsistent patient care, and difficulty with interpersonal interactions [11].

An important predictor of satisfaction within the team is team climate. Team climate is defined as the shared perceptions of the permanent or semi-permanent working group to which members are assigned [16]. A team is a working group, identified by its members, that interacts regularly to carry out their work [16, 17]. A positive perception of team climate has been found to increase team and patient satisfaction and decrease work-related stress [18]. West and Farr [19] proposed a model to explain team climate which includes four dimensions: shared goals and vision, participative safety, support for innovation, and task orientation.

Team member satisfaction is also enhanced by the presence of transformational leaders. Transformational leaders are leaders that emphasize interpersonal relationships, increase team member’s effectiveness [20–22], influence the beliefs and attitudes of their followers, and align members towards organizational success [21, 23]. In health care organizations, transformational leaders are recognized for their ability to facilitate change, increase job commitment and satisfaction, and improve patient outcomes [24–27]. The four dimensions of transformational leadership include motivation, individualization, idealized influence (or charisma), and intellectual stimulation [20].

To understand teamwork structure/processes like team climate and leadership, it is necessary to explore social networks. The social networks of a team describe the team’s patterns of communication, their professional associations, and relevance within the team [28, 29]. Teams need to share information to complete tasks and to develop a network of communication and social influences [30, 31]. Social network analysis (SNA) establishes that relationships are conditioned by the position occupied within the social structure and can be explained by analyzing the patterns of distribution of these positions and the networks that are formed [32].

The Chilean health system consists of public and private components and a competitive labor market between them. Both components need qualified health personnel to provide quality and safe care. Chilean Human Resources for Health (HRH) policies focus on professional gaps in the public sector specifically [33]. The Chilean Ministry of Health has worked to improve working conditions to attract and retain health care professionals. While there are a few Chilean studies regarding job satisfaction at the individual level [9, 34], none have focused on satisfaction among the interprofessional team as the unit of study.

The purpose of this study was to examine social exchanges, team climate, and transformational leadership as predictors of team member satisfaction in a hospital setting. We hope to inform what is known about hospital turnover and collaboration within interprofessional health teams. The long-term goal is to inform the HRH policies in order to increase professional satisfaction in teams, and improve access to public health services in Chile.

## Methods

### Research design

The study used mixed methods, sequential/explanatory design conducted in two phases [35, 36]. The findings of the quantitative and qualitative components were integrated to form meta-inferences and conclusions.

### Study site and sample selection

The setting was a 700-bed university hospital in Santiago, Chile, serving patients with private and/or public health insurance. The hospital has both high- and low-complexity units, and 1600 healthcare providers [e.g., 20% physicians, 31% register nurses, 3% nutritionists, 4% midwives, 4% physical therapists, and 38% nursing technicians] providing direct patient care. Interprofessional teams were the unit of analysis for the study. The participants were healthcare providers that had worked with the same team for a minimum of 6 months and shared patient care responsibilities. Students, administrative, and support staff were excluded from the study.

#### Population and sample

Using intentional sampling, the investigator recruited 409 team members grouped in 53 interprofessional teams. An interprofessional team was defined as the group to which professionals were assigned and in which they identified and interacted with, at least three times a week. An interprofessional team needed to include individuals from a minimum of two professions who worked together (e.g., sharing patients and a team leader) for at least 6 months. Beginning with nursing, register nurse and nurse technicians were asked to identify the teams they worked with. The researcher then asked the other professional groups (physician, nutritionist, midwives, physician therapist) if they identified themselves as being part of a team. If they agreed, they were then asked to identify their team. If they worked in multiple teams, they were instructed to choose their primary team.

### Phase I, stage 1

Phase I involved a descriptive correlational design to examine the relationships among team member satisfaction, team climate, and transformational leadership. Control variables included age, gender, profession or activity, time working for the hospital, their current team, and the number of team members. Data were collected between October 2015 and May 2016. Professionals completed the study instruments individually at a private setting within the hospital, and data were stored in a secure location. For the purpose of analysis, individual responses were grouped based on the interprofessional team with which they self-identified. Responses with greater than 20% missing data were excluded from analysis.

#### Study instruments

*Team member satisfaction* was measured using the instrument adapted by Gladstein [10] and validated in Spanish [11]. A previous study reported a Cronbach’s alpha of 0.80 [11]. The scale is a 5-item scale, using a Likert-type response from 1 (completely disagree) to 7 (completely agree) for each item. The questions assessed the extent to which the members of the team expressed satisfaction with colleagues (item 1), team processes (items 3 and 5), and results obtained (items 2 and 4). Higher scores indicated greater team member satisfaction. Cronbach’s alpha for this study was 0.94.

*Team climate* was measured using the Team Climate Inventory [16] a 14-item measure [37] validated in Spanish [38]. Previous studies reported a Cronbach’s alpha of 0.91. The measure uses Likert-type responses from 1 (completely disagree) to 5 (completely agree). Higher scores indicated a better or more desirable team climate. Cronbach’s alpha for this study was 0.93.

*Transformational leadership* was measured using the Multifactor Leadership Questionnaire [20] validated in Spanish [39]. A previous study reported a Cronbach’s alpha of 0.90. The 20-item scale uses Likert-type responses from 0 (completely disagree) to 4 (completely agree). Higher scores indicate greater perception of transformational leadership behavior. Cronbach’s alpha for the current study was 0.96. In addition, each participant was provided with the definition of a transformational leader and asked about the team member they perceived to be a transformational leader.

#### Data analysis

Quantitative data was entered into SPSS, version 22. Descriptive statistics were calculated including percentages, frequencies, and counts. Linear regression analysis was also conducted. The significance level was set at 0.05 for all statistical tests.

### Phase I, stage 2

**Fig. 1:**
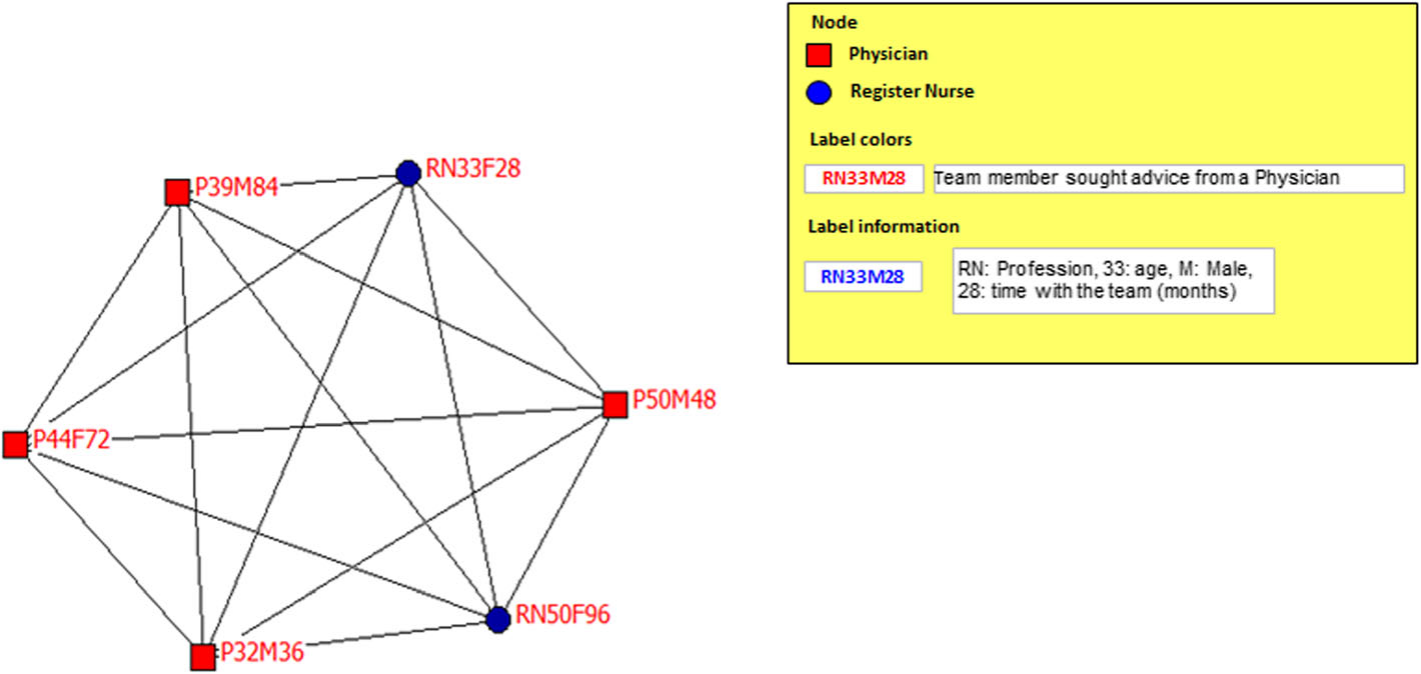
Interprofessional team with the highest team satisfaction: network for work advice

**Fig. 2:**
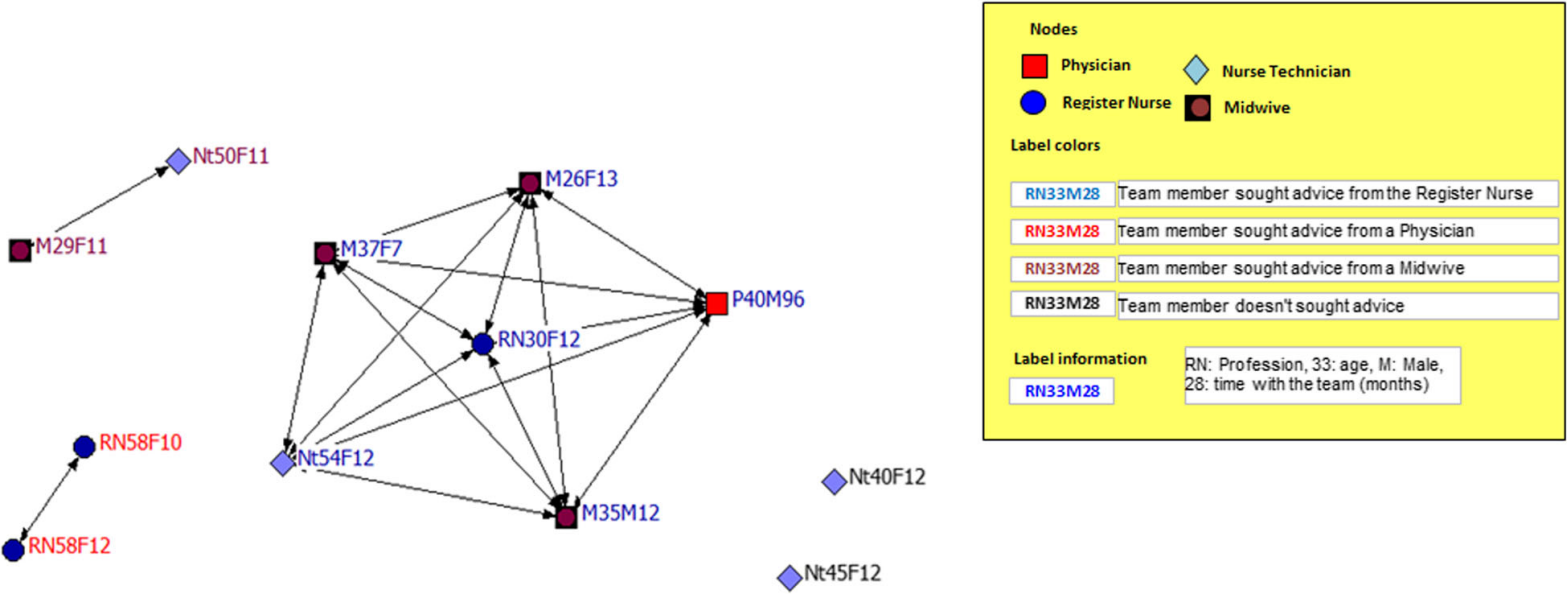
Interprofessional team with the lowest team satisfaction: network for work advice

**Fig. 3:**
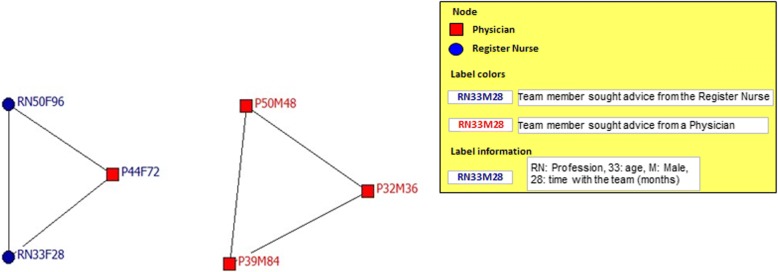
Interprofessional team with the highest team satisfaction: network for personal support/advice

**Fig. 4:**
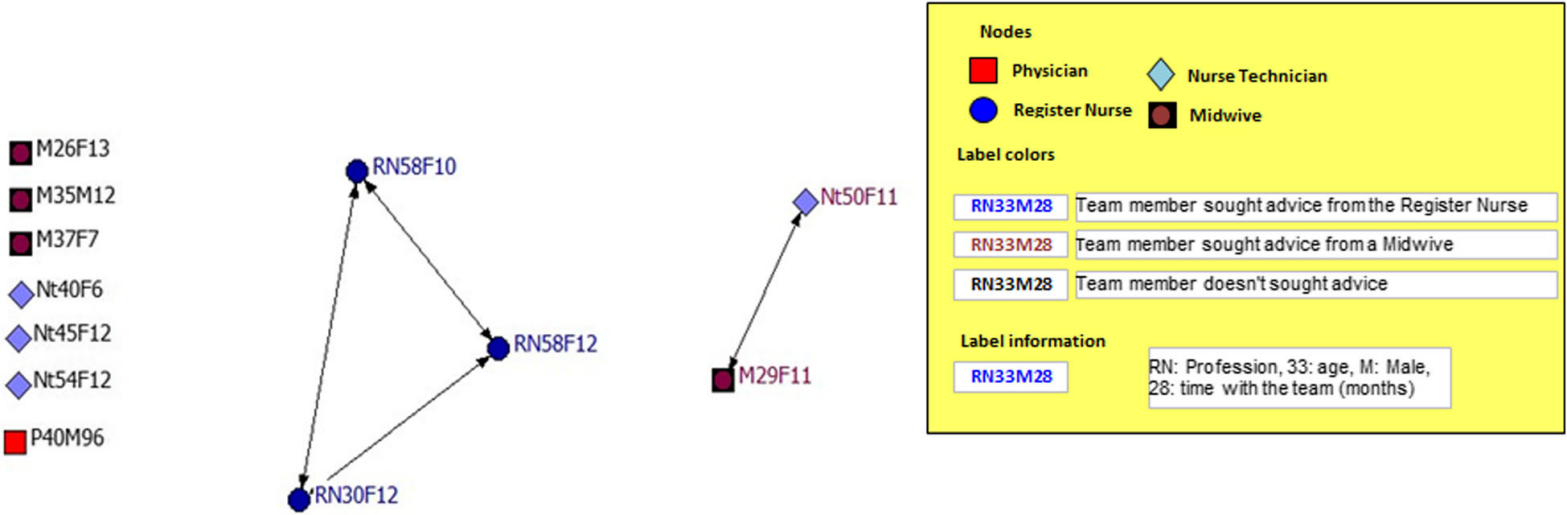
Interprofessional team with the lowest team satisfaction: network for personal support/advice

This stage involved social network analysis to identify interactions between team members from the interprofessional teams that reported the highest and lowest team satisfaction scores. Two previously used questions [32] were adapted and explored professional relationships within the team. The questions referred to work advice and personal support. The work advice question was (1) “Who do you go to when you have any need, difficulty or problem at work?” The personal support question was (2) “Who do you go to when you have a personal problem?” The answers to these questions allowed the researchers to calculate several measures including (1) density of the whole network (team) represented by the number of interactions (represents by loops) between professionals (represented by nodes) of the all possible connections (from 0 to 100%); (2) isolates, defined as the professionals that are separated or segregated from the rest of the team members; (3) centrality, defined as the profession that the majority of the team seek advice/support from (range 0 to 100%); and (4) subgroups of members connected between themselves within the team [32]. The graphical representation of the network (Figs. 1, 2, 3, and 4) included the profession (node) and a label indicating professional role, age, gender and time with the team. The color of the label indicates the profession whom each member sought work advice or personal support from (e.g., a red label on a node of the profession indicates that members sought advice from a physician).

#### Data analysis

Sociometric network analysis was applied using standard procedures with UCINET-6 software for Windows.

### Phase II

Phase II involved semi-structured interviews to understand team satisfaction related to coordination of clinical work, patterns of interpersonal relations, and communication among professionals [40]. Interviews were conducted with team members who reported either high or low satisfaction scores. The semi-structured interview guide was based on the theoretical references underlying the variables of interest.

#### Data analysis

Thematic content analysis was used to develop inferences about the topics of the study [40]. An inductive and deductive approach was used to examine the interviews for words, concepts, and themes. This approach allowed for identification, indexing, and retrieval of relevant content [40]. NVivo software was used for the analysis and the rigor control followed criteria outlined by Guba [40].

### Phase III

Phase III involved integration of the results from phases I and II, using an interactive process in which the results of both stages were analyzed within the framework of the theoretical references. The quantitative results were interpreted and explained using the qualitative results. Finally, the research team reviewed and agreed upon the summary for each category [41, 42].

#### Ethical, consent, and permissions

The Ethical and Scientific Committee of the participating institution (Faculty of Medicine, Pontifical Catholic University of Chile—protocol #15-059) approved the project. Written and verbal informed consent were obtained from participants before each stage of data collection.

## Results

### Results from phase I, stage 1

A total of 409 health professionals grouped in 53 interprofessional teams were identified (Table 1).

**Table 1:** Team member’s characteristics and role within the interprofessional team

	Count	Percent	Mean	SD
	409	100		
Sex				
Female	343	83.9	_	_
Male	66	16.1	_	_
Team role			Team conformation	
Nurse technician	190	46.5	43.40	18.38
Register nurse	132	32.3	32.87	15.27
Physician	45	11.0	14.23	16.67
Physical therapist	18	4.4	4.27	6.81
Midwife	12	2.9	2.39	7.84
Nutritionist	12	2.9	2.84	5.87
Care complexity units				
High-complexity wards	35	66	_	_
Low-complexity wards	18	33	_	_
Age			34.9	5.3
Time working in the hospital (in months)	_	_	99.76	105.47
Time working with the team (in months)			42.6	42.3
Number of team members			7.7	3.4

Register nurses and nurse technicians were found in 100% of the interprofessional teams, and 83.9% (*n* = 343) of the team members were women. Physical therapist, midwives and nutritionists comprised less than 10% of the professionals on teams. The majority of the team members (70%) were younger than 38 years of age. Health professionals reported that the length of time working in the hospital ranged from 6 to 504 months and the time working with the team varied from 6 to 240 months. Variables were normally distributed; therefore, parametric tests were used for all subsequent analysis (Table 2).

**Table 2:** Interprofessional team member satisfaction, team climate, and transformational leadership

	Teams	Mean	SD	Min/max
Satisfaction	53	22.71	3.05	11.5/28
Team climate	53	54.13	5.62	40/69
1. Team objective	53	16.71	1.37	13.6/20
2. Participative safety	53	15.36	1.88	10.2/19.8
3. Task orientation	53	11.27	1.40	7.8/14.8
4. Support for innovation	53	10.79	1.42	7.3/14.3
Transformational leadership	53	65.67	7.19	41/77.3
1. Idealized influence (charisma)	53	26.99	2.80	17.3/31.7
2. Inspiration motivation	53	13.10	1.51	8.3/16.0
3. Intellectual stimulation	53	12.67	1.59	6.8/15.6
4. Individualized consideration	53	12.93	1.75	8.8/16.0

Mean satisfaction of the study participants was 22.7 (SD = 3.04, range 11.5–28.0). Teams that reported higher and lower satisfaction scores varied on variables, including composition and length of time working together. The team with maximum satisfaction worked as specialty consultants, included six members [physicians (3) and register nurses (3)], and reported working together for over 60 months. In turn, the least satisfied team worked on a high-complexity unit and had 12 members [midwives (4), register nurses (3), nurse technicians (4), and physician (1)]. This team worked together for 13 months, except for the physician with 96 months. This team also had the lowest team climate and transformational leadership scores. Participants had a mean score of 54 (SD = 5.62) on the team climate measure. The team with the highest team climate score worked in an oncology unit and had four members [register nurse (1), nurse technicians (2), and physician (1)]. Their average time working together was 13 months.

At the individual level, the register nurse had the highest transformational leadership score (mean score = 64), followed by the physician (mean score = 33). At the team level, the mean score of the transformational leadership measure was 65.6 (SD = 7.1). The team with the highest score worked on a medical-surgical unit with 5 members [register nurses (2), nurse technicians (2), and nutritionist (1)] and worked together for 33 months.

Transformational leadership and team climate were entered into a linear regression model, and both were found to be predictors of team member satisfaction. The overall model was significant (*F* = 29.12, *p* < 0.005), and the adjusted *R*^2^ was 0.75 (Table 3).

**Table 3:** Logistic regression predicting interprofessional team member satisfaction

Variables	*β*	Std. Error	*t*	*p*	95% CI for *β*	
					Lower bound	Upper bound
Transformational leadership	0.17	0.049	3.416	0.001	0.069	0.267
Team climate	0.26	0.061	4.237	0.000	0.136	0.382

Satisfaction adjusted *R*^2^ = 0.756. *CI* confidence interval. Dependent variable: team member satisfaction score *p* > .005. Goodness of fit: Akaike (AIC) 206

The model explained 75% of the variance in the outcome variable of team member satisfaction. Each unit increase in transformational leadership score and team climate score resulted in 0.17 (95% CI, 0.077 to 0.259) and 0.26 (95% CI, 0.146 to 0.372) unit increase in team satisfaction scores, respectively (Table 3). With regard to demographic characteristics, only time on the team had a significant association with team satisfaction.

### Results from phase I, stage 2

Social network analysis comparing the network for work advice and personal support between the most and the least satisfied interprofessional teams revealed that the work advice network of the team with the highest satisfaction (Fig. 1), formed a close and highly cohesive network (100%) with all its members [register nurses (3) and physicians (3)] directly connected to each other (100%). This team received work advice from a physician. The least satisfied team [midwives (4), register nurses (3), nurse technicians (4), and physician (1)] represented a fragmented network for work advice (Fig. 2). This team showed a density of only 25%, having one subgroup, two pairs, and two isolated (not connected to another team member) members. The biggest subgroup had six members seeking advice from a register nurse, two members looking to the physician for advice, and two seeking advice from a midwife. Two nurse technician appear isolated from the team. The subgroup who sought advice from register nurse had the highest centrality (36%).

The social support of the team with the highest satisfaction (Fig. 3) showed minor density/cohesion (40%) divided between two subgroups. The first subgroup was connected around the register nurse and included two register nurse and one physician. The second subgroup included three physicians seeking advice from a physician. These two subgroups shared centrality of the network by 50% each. The social support network of the least satisfied team (Fig. 4) had a density of just 9%. There was one small subgroup of three register nurses, who sought support from themselves and centralized 18% of all interactions.

### Results from phase II

Fifteen interviews were conducted with professionals belonging to four interprofessional teams with the highest and lowest team satisfaction scores. The health professions who participated in the interviews included three physicians, five nurse technicians, two nutritionists, two physical therapists, and three register nurses. Using content analysis, the research team noted 16 categories represented by narrative codes, which generated six themes (Table 4).

**Table 4:** Characteristics of interprofessional team work that foster team satisfaction

Themes	Categories supplemented by team members narratives
1. Attributes of interprofessional team work	Interprofessional team members recognize the meaning of delivering **patient-centered** **care** with a **common goal** over individual differences: One nutritionist recognized “We paddle for the same side.” A nurse technician explains “people find it hard to under stand that after all, they have to work together! They have to do it! to focus on the patient, not on their relationships. If they get along or get along badly” and a register nurse and nurse technician appears as a **close task team**, a physical therapist expresses it*….* “I’m not in so many meetings ... nursing is the strongest task nucleus!!”
2. Collaboration, communication and social interaction	Team members emphasized the need for opportunities and places for **interaction** **and cordial communication** that facilitates interprofessional teamwork relations. A nutritionist expresses “It helps communication, empathy, in general (…) super good experiences! Even with some physicians. There is a personal relationship, then the work becomes much easier.” Team members recognize that **professional horizontality** facilitates the work and collaboration, a register nurse “everyone fulfills a task and all the work is valuable.” A nurse technician indicated “physician are visitors to the units, the ones together every day are nurse technicians, register nurse, physical teraphists.” Although for some physicians, team is there to facilitate his performance “You have to get support from your nurse, your physical therapist and even your nurse technician, because they spend more time with the patient.” **Recognizing one’s own and others’ roles strengthens the synergy**, facilitated by the permanence in the interprofessional team*.* One Physician stated “we need to be clear about the role, value of that role and empower it and give it the importance that corresponds!”
3. Interprofessional team innovation	To **encourage opportunities to create** and promote new ways of doing things, a register nurse relates “in clinical practice, they are the ones! from them (team members) is born the solution to a problem. You have to listen to them because it also makes is easier to get their adherence.” This position can generate tension between **reform and resistance to change**, a physician exemplifies “we always did this! It is one of the things that disturbs me most.” Results highlight the **contribution of the younger generations**, as a register nurse stated “currently people are focused on the opportunities that one gives them. Sometimes there are very young people(…) who have very good ideas and work in shaping them as a project.”
4. Shared leadership	The interprofessional team expects from the leader **motivational communication**, sharing a vision of work with a meaning*.* The physician explains “when people feel motivated and welcome and the leader manages to convey the importance of the goal and of each person’s role to achieve it.” The nutritionist demands that “the supervisors do not sit with the power! Everybody does things.” A leader should consider **individual and collective** **well-being** by deepening in individual motivations. A physician expressed it as “simple things like greet them in the morning, that is enough.” A nutritionist suggests “ask them, how are they? the family? know if they are well.” The presence of a formal leader from the organization and a second one among team members rising when needed, has being classified as **situational and shared leadership**. A physician considers “is attributed a lot of leader role to the physician, being that probably does not have leadership role… all should be leaders in their work.” The informal leader is a **facilitator of dialog and communication**, a nutritionist indicates “there has to be a leader (…) no matter young, old, have to be a conciliatory person and know how to communicate with people(…) informal leaders have an added value.” Interprofessional team members recognized in the register nurse a leadership that facilitated their relationship with the physician. One physical therapist expressed “they know everything that happens, they do not have problem in speaking strong, in approaching a physician. Many other people panic about talking to a physician.”
5. Interpersonal relationship interface work/social	Team members perceived the interpersonal relations on two levels, one level focuses on work and the other level focuses on social relations. Both levels were noted to enhance **climate of confidence and security**. A nutritionist stated “it becomes much easier when you have an interpersonal relationship, more than just work(…) there is more confidence to ask.” Another physician added “you have your team to trust, 100 % in what your people are doing.” **Dialog and interactions** that consider the other are indispensable. One physical therapist insisted “it is super important! No matter that I don’t like her or if she is my friend or not, but if I know what the other does I will respect it.” Register nurse is central in facilitating the **mediation** **of the information** with the physician. A nutritionist explains “the nursing staff, invite us to know new things! so that we all handle the same information.” Another nutritionist adds “several times want to talk to (the physician) and the register nurses handle all the telephone numbers.”

### Results from phase III

Table 5 displays the integration of the three data sources. It identified characteristics that emerged as underpinnings of interprofessional team member satisfaction. The high level of concordance across the sources is illustrated.

**Table 5:** Mixed methods integration to explain team member satisfaction

Quantitative results	Qualitative results
Interprofessional team composition 50% include register nurse, physician and nurse technician. 33% include midwives, physical therapist, register nurse and nurse technician.	Task core represented by register nurse and nurse technician. Uniprofessional teams of physical therapist and nutritionist, but close to the register nurse Participation of physician on teams mostly to deliver indications
Team climate (M: 54.13, SD: 5.3) 1 point increase on team climate increase team satisfaction by 0.23 point	Facilitates team member satisfaction: 1. Patient-centered care 2. Common objective over individual differences 3. Cordial interaction and respectful communication facilitating the establishing of trust. 4. Recognition of one’s own role and the other team members. 5. Recognition of the fair value of individual contributions 6. Empower innovation and generational participation.
Register nurse is recognize by 75% of team members as the transformational leader of interprofessional teams. Transformational leadership (M: 65.67, SD: 7.19) 1 point increase on transformational leadership increase team satisfaction by 0.20 point.	Recognized by team members because of the following: 1. Considers individual and collective well-being in Interprofessional Teams. 2. Know and recognize the talents and abilities of others 3. Interested in the personal dimension of the person 4. Generate bonds of trust through formal and informal instances.
Differences between higher and lower team satisfaction in network for work advise density (100–25%) and centrality (100–36%) respectively. In network for personal support/advise density (40–9%) and centrality (50–18%), the same tendency was observe.	Facilitates team member satisfaction: 1. Having a close task core 2. Professional horizontality to share information. 3. Interactions and communication that include a personal dimension. 4. Centrality of the relations relatives to having common objectives.

## Discussion

The findings of this study revealed that 409 health professionals perceived themselves as working on interprofessional teams from a total of approximately 1600 health professionals. Register nurses and nurse technicians were present on all of the teams [2, 43]. Interprofessional teams were most often found in wards/units where the complexity of patient care demanded collaborative work, or patients needed specialized care that required the presence of an expert consultation team (e.g., diabetes and ostomy) [44, 45]. Phase I results revealed that team member’s satisfaction, transformational leadership, and perceived team climate were significantly associated. Studies in clinical settings have identified similar results at the individual level, especially transformational leadership, team climate, and overall satisfaction [4, 17, 21, 25].

This study found that team climate was a better predictor of team satisfaction as compared to transformational leadership. The length of time working with an interprofessional team was also associated with satisfaction. Given that health care organizations in Santiago tend to rotate health professionals among different teams as a way to broaden their skills, these findings are noteworthy. A recent study found that a group’s life cycle stage is a relevant variable to achieve team satisfaction [46].

Findings from work advice network of the most satisfied team are worth noting. Results suggest that when a team structured itself around one professional, this allowed its members to approach and be approached easily, and facilitated information exchange through the network effortlessly. Teams with the least satisfaction revealed a fragmented structure with members organized as subgroups. These subgroups depended on each other for information and acted as mediators of communication flow. In terms of decision-making and sharing information, the incidence of pairs, some of which were isolated, represented a dangerous situation. Members of these teams did not agree regarding the best work advisor for assistance in delivering care to their patients. The organization of social support networks was even more fragmented, with half of them being isolated from the rest of the team. Other studies have used social network analysis to design quality improvement teams [28], to compare measures of friendship and work network [47], to improve workflow, and to better understand relationships [48].

The qualitative phase showed that common goals and patient-centered care are crucial characteristics of satisfied teams. Results emphasized the importance of interactions between members. Furthermore, professionals need to recognize the contribution of each team member and to know the responsibilities of all team members. Participants recognized an interface of reciprocity between personal and professional relationships. Other studies have reported similar results [4, 26, 43, 49, 50].

Team members’ perception of leadership is based on their interpersonal relationships with the leader. Integration of findings from both phases revealed that health professionals’ satisfaction with their interprofessional team was associated with characteristics of the team (e.g., location and permanence) and whether the team facilitated interpersonal relationships. Important aspects of the interpersonal relationships included cordial interactions, respectful communication, and shared decision-making. Similar results have been reported previously [15, 21, 22, 24, 43, 51–53].

Our findings indicated that team member’s satisfaction is associated with a team climate in which objectives/goals are shared. This type of team climate facilitates team members’ participation and task commitment. Positive interpersonal relationships involved recognition of equitable value of individual contribution to patient care and role clarity of all team members. These results are consistent with studies on HRH that recommend supporting positive interpersonal relationships and active listening, thereby facilitating interprofessional collaboration and teamwork synergy [6, 19, 34, 52].

Results of team climate for innovation highlight the contribution of the younger generation, through creativity and originality in the search for solutions. The literature recognizes the benefits of each profession’s talents and skills and encourages them to facilitate team identification and task commitment [30, 54]. Current research, focused on team climate for innovation, has documented tension between generations working together and resistance to change [54, 55].

Our results revealed the presence of shared (more than one) leader on interprofessional health teams. Recognized as a transformational leader, the register nurse was perceived as facilitating team member satisfaction through interpersonal relationships. This was accomplished through instances of dialog and genuine interest beyond professional boundaries. Other investigations on nursing teamwork support these results [56, 57]. A second team leader, most often a physician, was appointed by the organization and was task focused. Studies recognize this role as more traditional style of leader, one that gives direction with restricted communication and support [43, 58].

The analysis of team member’s professional and personal networks illustrated work associations and patterns of communication and information flow. The results also revealed an interface of influence between team members’ professional and personal networks. Positive interpersonal relationships can foster friendship and trust [23] particularly with jobs that require task interdependence and active collaboration [26]. Nonetheless, reduced interactions between professionals may lead to dissatisfaction, frustration, conflicts, and team fragmentation [11, 15].

This study had several limitations. First, the use of a cross-sectional design did not allow us to establish causality. A second limitation was the use of an intentional sample, which limits generalizability of findings. Another limitation was social desirability bias, which may have resulted in respondents over/under reporting interpersonal relationships or team leadership. We encouraged frank responses during survey administration to reduce social desirability bias. A fourth limitation was recall bias, and the accuracy of the data collected may have been influenced by this type of bias. Despite these limitation, the study had several strengths. The novel attempt to study this phenomenon in Chile and the use of mixed methods were strengths of the current study. Mixed methods generated a rich description of interprofessional work practice and allowed us to move beyond descriptive to explore team member satisfaction.

## Conclusion

This study provided a comprehensive approach to describe the satisfaction experienced by members of interprofessional teams in clinical contexts. Team climate was dependent on interactions around a shared goal, which in turn facilitated collaborative work beyond personal differences, and explained to a greater extent the satisfaction within the team. Recognition of individual contributions to patient care was facilitated by members’ permanence on the team. A recurrent theme was the need for clarity of professional roles and definition of responsibilities.

Team leadership was shared between the designated leader from the organization and the transformational leader recognized by team members. The recognition of the interdependence between the professional and personal dimension of the team encouraged integrated teamwork and should be considered in HRH strategies. Analysis of social networks allowed the investigators to observe patterns of communication and shared information as a way to solve work and personal problems within teams.

Our research results have the potential to contribute to the planning and decision-making in the field of HRH, providing elements to promote teamwork and its management as well as supporting team member satisfaction. In turn, this could lead to job permanence especially where the local needs are more urgent (Chilean public health sector). Our results are also aligned with the Global Strategy for Human Resources for Health (HRH) 2030 that call to strengthen interprofessional teamwork collaboration. Our paper highlights central elements of team climate and leadership that enhance members’ satisfaction within the team. This knowledge can be used to develop strategies that limit health professional turnover and help to meet the growing and complex needs of health users. Future research should focus on barriers to teamwork, deepening the understanding of the interface between the professional and personal dimension of HRH and its impact on work results. Finally, these results need to be validated studying other types of interprofessional teams to determine their level of transferability to other teams and contexts.

## Abbreviations

HRH: Human Resources for Health
MINSAL: Chilean Ministry of Health
WHO: The World Health Organization
